# Study of all-group-IV SiGeSn mid-IR lasers with dual wavelength emission

**DOI:** 10.1038/s41598-023-45916-4

**Published:** 2023-10-28

**Authors:** Grey Abernathy, Solomon Ojo, Abdulla Said, Joshua M. Grant, Yiyin Zhou, Hryhorii Stanchu, Wei Du, Baohua Li, Shui-Qing Yu

**Affiliations:** 1 Department of Electrical Engineering, University of Arkansas, Fayetteville, AR 72701 USA; 2 Material Science & Engineering Program, University of Arkansas, Fayetteville, AR 72701 USA; 3 Institute for Nanoscience and Engineering, University of Arkansas, Fayetteville, AR 72701 USA; 4 Arktonics, LLC, 1339 South Pinnacle Drive, Fayetteville, AR 72701 USA

**Keywords:** Solid-state lasers, Semiconductor lasers

## Abstract

Direct band gap GeSn alloys have recently emerged as promising lasing source materials for monolithic integration on Si substrate. In this work, optically pumped mid-infrared GeSn lasers were studied with the observation of dual-wavelength lasing at 2187 nm and 2460 nm. Two simultaneous lasing regions include a GeSn buffer layer (bulk) and a SiGeSn/GeSn multiple quantum well structure that were grown seamlessly using a chemical vapor deposition reactor. The onset of dual lasing occurs at 420 kW/cm^2^. The wider bandgap SiGeSn partitioning barrier enables the independent operation of two gain regions. While the better performance device in terms of lower threshold may be obtained by using two MQW regions design, the preliminary results and discussions in this work paves a way towards all-group-IV dual wavelength lasers monolithically integrated on Si substrate.

## Introduction

Silicon based high performance lasers have long been desired for Si photonics. Due to the indirect bandgap nature of Si, group III-V materials such as GaAs and InP, and group IV material Ge were grown on Si to build band-to-band lasers. Over the past several years, much progress has been made toward the growth of group IV GeSn alloy on Si substrates. The motivation stems from the fact that the bandgap of GeSn undergoes an indirect to direct transition when the Sn content increases above 6.5–11 at.% for relaxed and compressively strained materials^1–3^. Material growth studies revealed that GeSn materials with high Sn fractions can be achieved under non-equilibrium growth conditions, such as using a chemical vapor deposition (CVD) reactor at relatively low temperature (~ 300 °C) to overcome the low (~ 1.1 at.%) miscibility limit of Sn in Ge^4^. Recently, direct band gap GeSn semiconductors with Sn composition as high as 18 and 22.3 at.%^5–8^ and room-temperature optical emission down to 0.36–0.39 eV^5^ have been demonstrated. This makes GeSn alloys viable optical-gain materials for the realization of high-efficient and cost-effective near and mid-infrared lasing sources on the Si platform.

Following the experimentally identified direct bandgap of GeSn^9^, the first optically pumped GeSn laser on Si was demonstrated with Sn concentrations of about 12.6 at.% up to 90 K^3^. Afterwards, many research works were conducted to improve the lasing performance. Higher lasing temperatures up to 270 K were obtained using the double heterostructure (DHS) Fabry–Pérot cavity^10–12^, followed by room temperature lasing demonstrated using micro-disk structures^13,14^.

In parallel to developing the GeSn bulk laser, GeSn/SiGeSn multiple quantum well (MQW) structures were studied to reduce the lasing threshold. The Si and Sn compositions in barrier and well can be engineered to form type I band alignment^15,16^, which leads to effective confinement of both electrons and holes in the QW resulting in enhanced radiative recombination rates. Moreover, tuning the width of the well provides additional control over the emission wavelength and barrier height. It is worth mentioning that for GeSn QW structure design, band structure calculations and photoluminescence (PL) studies revealed that type I band alignment and direct bandgap QW are attainable when using a strain-relaxed GeSn buffer layer in addition to a Ge buffer^17^. This aids in mitigating the compressive strain of coherent growth, and therefore enhances band gap directness of the GeSn QW. The design and lasing properties of GeSn/SiGeSn MQWs were studied by several research groups. For example, Stange et al.^18^ have compared MQW lasers with 22 and 12 nm QW thicknesses and have shown significantly reduced lasing thresholds for wider QWs as well as more than 10 times reduced threshold compared to bulk structures. Moreover, better performance in terms of lower threshold and higher lasing temperature was also reported by Margetis et al.^19^ In addition, Abernathy et al. have explored the thicknesses of the cap layer^20^ and of the active region^21^ and studied the optical confinement factor for the GeSn QW lasers. These results clearly indicated that the GeSn buffer plays an important role to deliver a high-quality QW stack.

It has been reported that a semiconductor laser being able to simultaneously emit dual wavelength has numerous potential applications such as differential absorption spectroscopy, interferometry, and generation of terahertz radiation. A dual-wavelength GeSn laser operating above 2 μm is additionally intriguing for light detection and ranging (LIDAR) in the eye-safety wavelength region^22^. Laser arrays emitting at different wavelengths have been demonstrated by many research groups as a viable approach for multi-wavelength lasing^23^. Moreover, multi-wavelength lasing from a single structure enables the realization of reduced Size, Weight and Power (SWaP), which is highly desired for the development of photonic integrated circuits. The key factors to achieve dual lasing in a single device are: i) two emitting regions and the partition layer that can be grown seamlessly; and ii) each region can provide sufficient net gain. Currently reported lasers employ two sets of QW regions with one set of shorter wavelength QWs and another set of longer wavelength QWs partitioned by a thin electrical barrier^24–26^, so that the population inversion can be satisfied in two regions simultaneously. In this work, we observe dual-wavelength SiGeSn lasing in the mid IR range under optical pumping. It is worth noting that a unique laser design was employed: one gain region consists of a SiGeSn/GeSn MQW stack while the other gain region is a GeSn bulk layer. From material growth perspective, the GeSn bulk layer was grown on Ge buffered Si substrate, which also serves as an additional buffer layer. The first SiGeSn barrier on top of GeSn buffer acts as partition layer. The two wavelengths correspond to emissions from the GeSn buffer layer and the MQWs region. Simultaneously lasing occurs under the pumping density of 420 kW/cm^2^ at the wavelengths of 2187 nm and 2460 nm.

## Materials and methods

### Material growth

**Figure 1:**
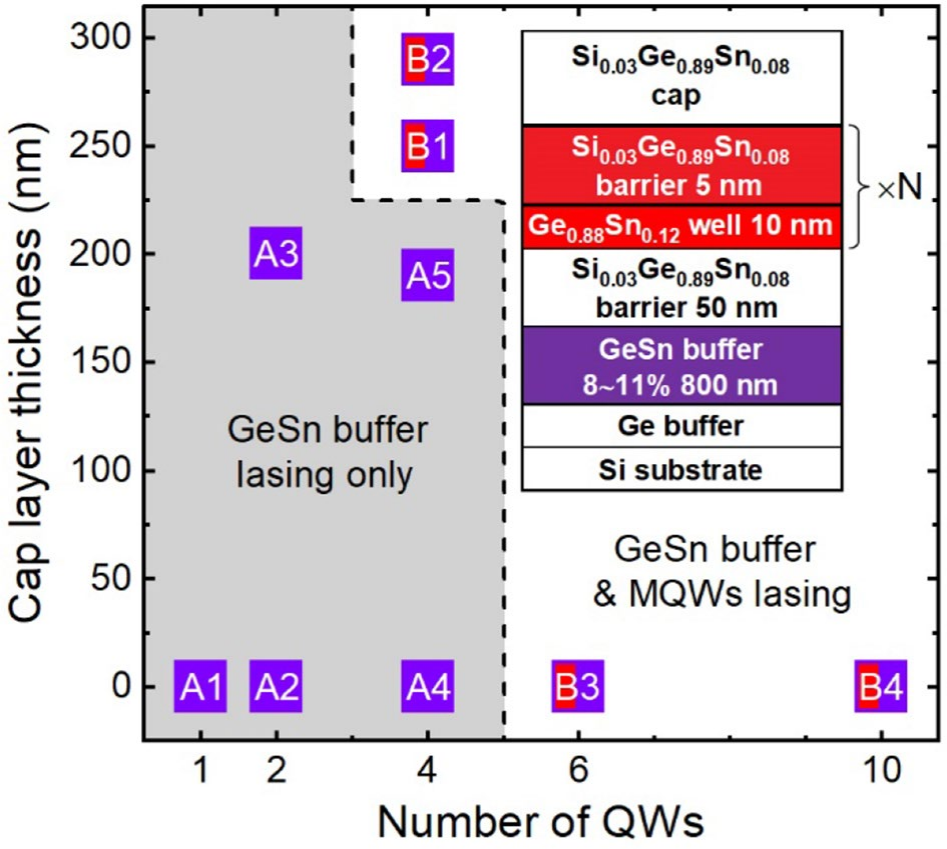
Sample design diagram showing the number of QWs and the cap layer thicknesses. Inset is the schematic cross-section of samples.

Two groups of samples were studied as shown in Fig. 1. Samples in group A have 1–4 wells, with two samples capped with relatively thin SiGeSn. Only buffer lasing was observed in group A samples. Group B includes two 4-well samples with relatively thick SiGeSn cap, one 6-well, and one 10-well samples. Dual-wavelength lasing was obtained for group B samples. The lasing behavior will be discussed in the next section. All samples were grown using an industry-standard CVD reactor with low-cost commercially available precursors for Si, Ge, and Sn, respectively^27^. First, a ~ 1 μm thick Ge buffer layer was grown on Si(001) substrate by a two-step growth method, at low and high temperatures, to promote layer-by-layer growth and ensure high material quality^28^. The growth was followed by the deposition of a nominal 800-nm-thick Ge_1-x_Sn_x_ buffer. Due to the strain-relaxation enhancement (SRE) of Sn incorporation^8,29^, the Sn fraction in the Ge_1-x_Sn_x_ buffer was increased from 8 to 11% during the growth. Afterward, a 50-nm-thick Si_0.03_Ge_0.89_Sn_0.08_ bottom barrier layer was grown on top of the Ge_1-x_Sn_x_ buffer, on which the MQWs stack with a 10-nm-thick Ge_0.88_Sn_0.12_ well and 5-nm-thick Si_0.03_Ge_0.89_Sn_0.08_ barrier was grown (see Fig. 1 inset). Several samples were covered with a varying thickness Si_0.03_Ge_0.89_Sn_0.08_ cap layer. The design diagram of all samples is shown in Fig. 1.

### Material and optical characterization

After the growth, the elemental composition, thickness, and strain values were determined by secondary ion mass spectrometry (SIMS) and X-ray diffraction (XRD) ω/2θ scans and reciprocal space maps (RSMs). The XRD measurements were performed using Philips X’pert MRD system equipped with a standard four-bounce Ge(220) monochromator, 1.6 kW Cu K_α1_ X-ray tube, and a Pixel detector. The PL measurements were performed using a standard off-axis configuration with a lock-in technique (optically chopped at 377 Hz) and 1064 nm excitation. The laser power was measured to be 20 mW. The PL emission was collected by a spectrometer and then sent to a PbS detector with a cutoff at 3.0 μm.

### Optical pumping measurements

Lasing was demonstrated for Fabry–Perot (F-P) ridge waveguide devices (0.2 mm width by 2.2 mm length) that were fabricated using the standard photolithography, wet-etching, and lapping processes. The fabrication details were reported previously^21^. Laser operation was achieved under optical pumping using a 1064 nm pulsed laser focused on a stripe using a cylindrical lens. A continuous flow cryostat was used for low-temperature measurements.

## Results and discussion

**Figure 2:**
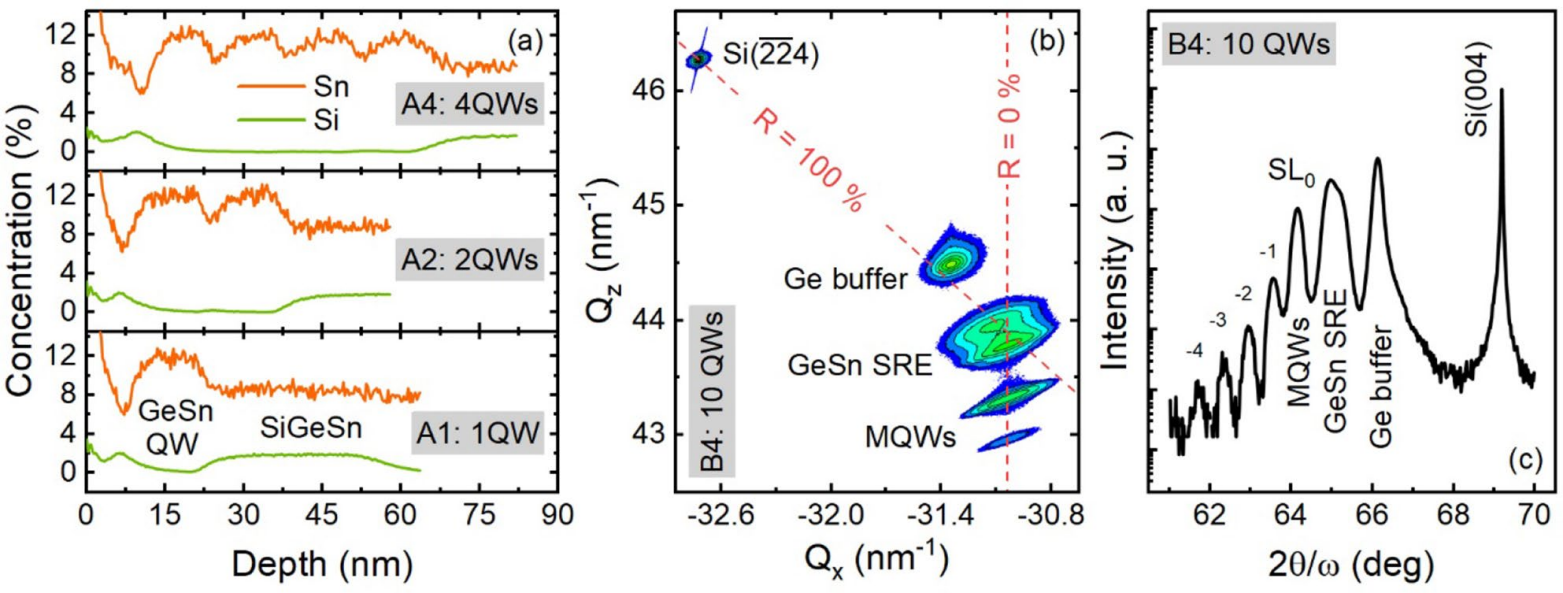
Structural characterization of MQWs samples. (**a**) The SIMS profiles of samples A1, A2, and A4 showing the Sn and Si compositions. (**b**) The XRD ($\overline{2 }\overline{2 }4$) RSM of sample B4 showing the coherent growth of MQWs on strain-relaxed GeSn SRE and (**c**) The ($004$) 2θ/ω scan displaying satellite peaks (SL_n_) related to the periodicity of the ten-period MQWs structure.

Material characterization was performed via high-resolution XRD and SIMS measurements. The typical SIMS profiles of samples A1, A2, and A4 are shown in Fig. 2a. The elemental distribution with depth reveals 9.4 ± 0.9 nm and 5.3 ± 1.1 nm wide QWs and barriers, respectively. Note that some degraded interface abruptness between well and barrier was observed. This is due to interdiffusion during the sample growth, which was investigated in reports elsewhere^30^. With such interdiffusion, the thicknesses of well and barrier are slightly wider. Figure 2b shows a typical XRD RSM of sample B4 which reveals strain-relaxed Ge and GeSn buffer layers located on the R = 100% line. The MQW’s peaks are vertically aligned along the R = 0% line with the GeSn buffer peak, indicating a pseudomorphic growth of the Ge_0.88_Sn_0.12_ QWs under -0.75% strain. In addition, the 2θ/ω XRD pattern of sample B4 (Fig. 2c) shows a series of satellite peaks (SL_n_), which reflects the well-barrier periodicity of the ten-period MQW structure. Usually, for an MQW stack there are no separate peaks for well and barrier. Instead, there is a zero-order peak (SL_0_) corresponding to the average lattice parameter of the well and barrier. The peaks at 66.2°, 65.1°, and 64.3° correspond to the Ge buffer, GeSn buffer, and MQW stack, respectively. The peak of the bottom SiGeSn barrier mostly overlaps with GeSn buffer peak, and therefore cannot be individually identified. The distance between the satellite peaks corresponds to a QW/barrier period of 17.2 ± 0.5 nm, which is in close agreement with the 14.9 ± 2.2 nm value obtained from SIMS.

**Figure 3:**
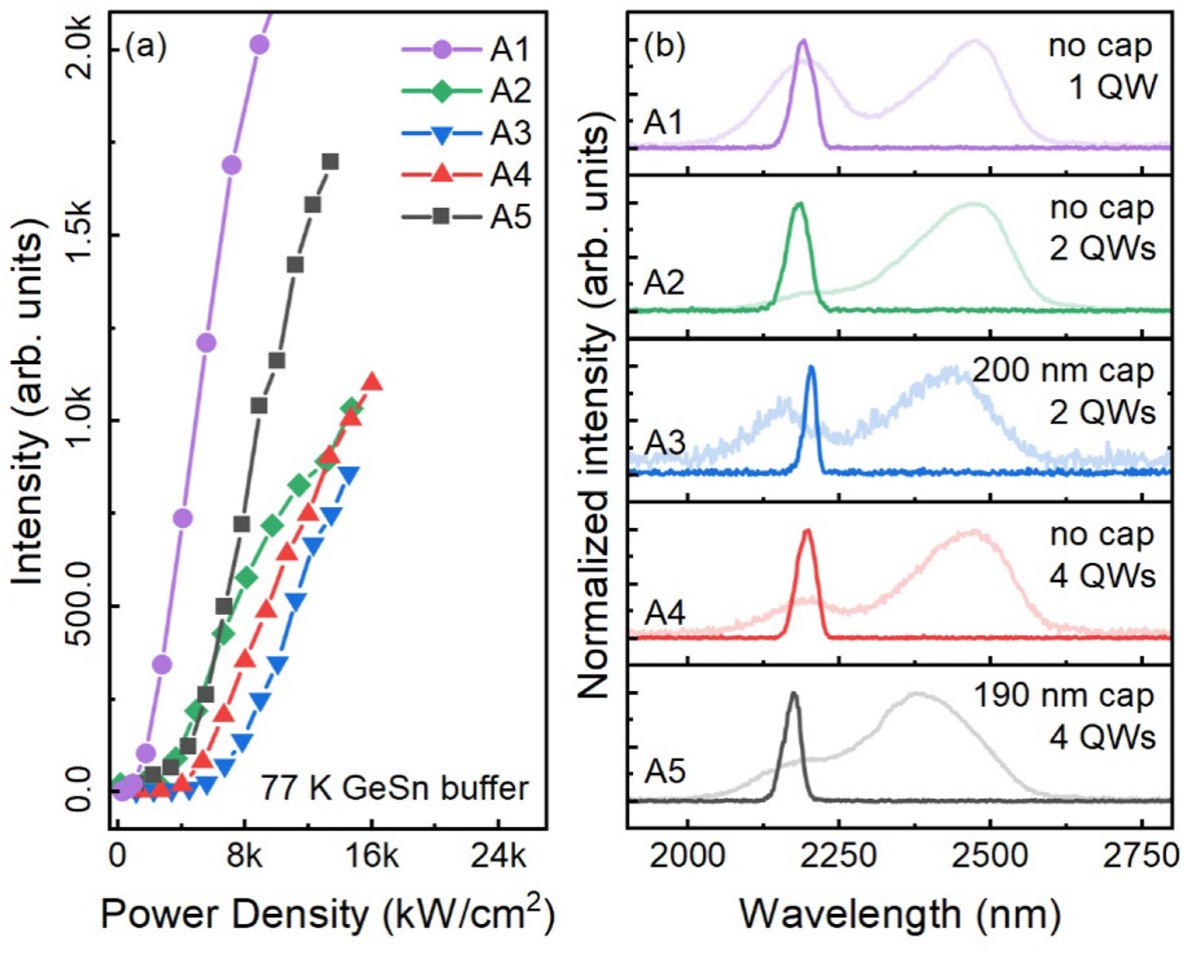
(**a**) L-L curves of group A samples. (**b**) Lasing spectra of group A samples showing lasing peaks at identical position, indicating the emissions are originally from the GeSn buffer. The background curves are PL spectra taken at 10 K.

The lasing characteristics of group A samples are shown in Fig. 3. Our previous studies^20,21^ indicate that without a cap layer, lasing cannot be achieved with single-, double-, and 4-well devices due to relatively small optical confinement factor. For samples A3, A4, and A5, although a ~ 200-nm-thick cap improves the optical confinement factor, it is still insufficient to reach threshold. On the other hand, the almost relaxed GeSn buffer is a direct bandgap material, and the 800-nm layer is thick enough for light absorption. With sufficiently high pumping power, lasing condition can be satisfied. Figure 3a shows L-L curves measured at 77 K of group A samples. Lasing threshold characteristics were clearly obtained for each sample. Since the emission is from the GeSn buffer layer, which is buried underneath the QW stack, the thicker QW region would lead to the higher threshold due to the top layers’ absorption. This trend can be clearly seen from samples A1, A2, and A4, having single, double, and 4 wells, all without a cap layer. Using a longer wavelength pumping laser could enhance the light absorption in the GeSn buffer layer, and consequently reduce the threshold. For instance, significantly reduced lasing thresholds were obtained under 1950 nm pumping laser compared to under 1064 nm pump laser according to our previous study^12^. Apart from that, the optical confinement in the MQWs can be improved by utilizing the micro-disk cavity approach, as recently demonstrated for GeSn heterostructure micro-disk laser^18,31^. Sample A3 has an even higher threshold because of a 200-nm-thick cap layer. Note that the threshold of sample A5 deviates from the trend. This is explained by the slightly higher material quality of the GeSn buffer layer compared to other samples. The detailed lasing threshold and temperature are summarized in Table 1.

**Table 1:** Threshold values at 77 K and maximal lasing temperature for GeSn buffer and MQWs lasing for all samples.

Sample	QWs number	Cap thickness (nm)	GeSn buffer lasing		MQWs lasing	
			Maximal temperature (K)	Threshold at 77 K (kW/cm^2^)	Maximal temperature (K)	Threshold at 77 K (kW/cm^2^)
A1	1	–	90	1650	–	–
A2	2	–	110	2770	–	–
A3	2	200	90	6800	–	–
A4	4	–	110	4700	–	–
A5	4	190	110	4500	–	–
B1	4	250	–	–	77	214
B2	4	290	–	–	77	664
B3	6	–	–	–	77	182
B4	10	–	–	–	90	267

Lasing spectra of group A samples at 77 K are shown in Fig. 3b. The curves are stacked for clarity. The PL background is also shown for comparison. Due to the stronger light absorption in the MQW region compared to the GeSn buffer, the MQW emission dominates the PL spectra, whose wavelength locates at 2400 ~ 2500 nm matching with bandgap estimation. It can be seen that all lasing peaks are at 2187 nm, also matching with bandgap estimation. This confirms that the emissions are from GeSn buffer: although QW regions are different for each sample, their GeSn buffers are almost identical in terms of Sn composition and layer thickness, resulting in the same emission peaks. Note that since the GeSn buffer features compositionally graded Sn content ranging from 8 to 11% along the growth direction, the lasing emissions were mainly from the region near the 11% Sn layer. This can be explained by the stronger absorption in the 11% Sn layer as well as the carrier funneling effect. Due to the higher absorption coefficient of the 11% Sn layer, light absorption in the 11% Sn layer is higher than that of the 8% Sn layer. In addition, the graded Sn content leads to a tilted band edge, and the photogenerated carriers in the GeSn buffer tend to transport from the wider bandgap (8% Sn) to the narrower bandgap (11% Sn) region, which additionally contributes to carrier accumulation in the 11% Sn region.

**Figure 4:**
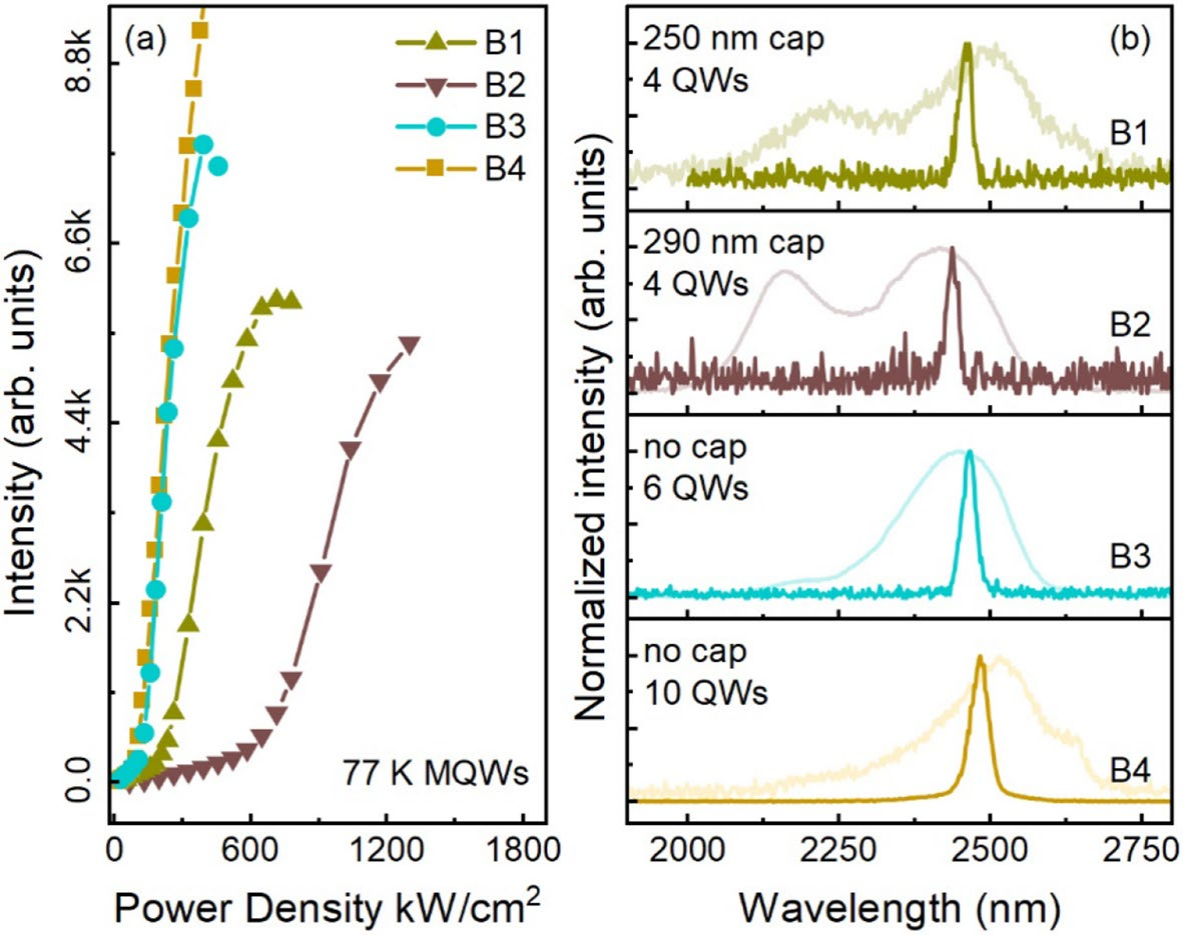
(**a**) L-L curves of group B samples. (**b**) Lasing spectra of group B samples showing that the emissions are originally from the GeSn QW region. The background curves are PL spectra taken at 10 K.

Figure 4a shows L-L curves measured at 77 K of group B samples. For all samples, the L-L curves exhibit a distinct threshold behavior, confirming the transitions from spontaneous emission to lasing operation with increased pumping power. For samples B1 and B2, having 4-wells with relatively thicker SiGeSn cap layers (250 and 290 nm) compared to samples A4 and A5 (0 and 190 nm), lasing is achieved due to higher optical confinement factor. Samples B3 and B4 show lower thresholds than B1 and B2. This is because of the increased number of wells that improves the net modal gain. The maximum operating temperature of 90 K is observed from sample B4 with 10-well structure. Figure 4b shows the stacked lasing spectra with PL as background, confirming the emissions are from MQW region. Note that for samples B3 and B4, the spontaneous emission from GeSn buffer disappears in PL spectra, indicating a strong light absorption in the MQW region. The lasing characteristics are summarized in Table 1.

In Table 1, the thresholds of GeSn buffer lasing are much higher than those of MQW lasing. This can be interpreted by the light absorption in the MQW region that “wastes” a large number of photogenerated carriers, and thus only a small portion of carriers can be contributed to stimulated emissions in the GeSn buffer layer. For optically pumped lasers, this issue can be alleviated by using a longer wavelength pumping laser that features longer penetration depth and therefore improves the absorption efficiency in the GeSn buffer. On the other hand, the maximum operating temperate of GeSn buffer lasing is higher than that of MQW. This is due to the bulk GeSn buffer features a higher optical confinement factor.

**Figure 5:**
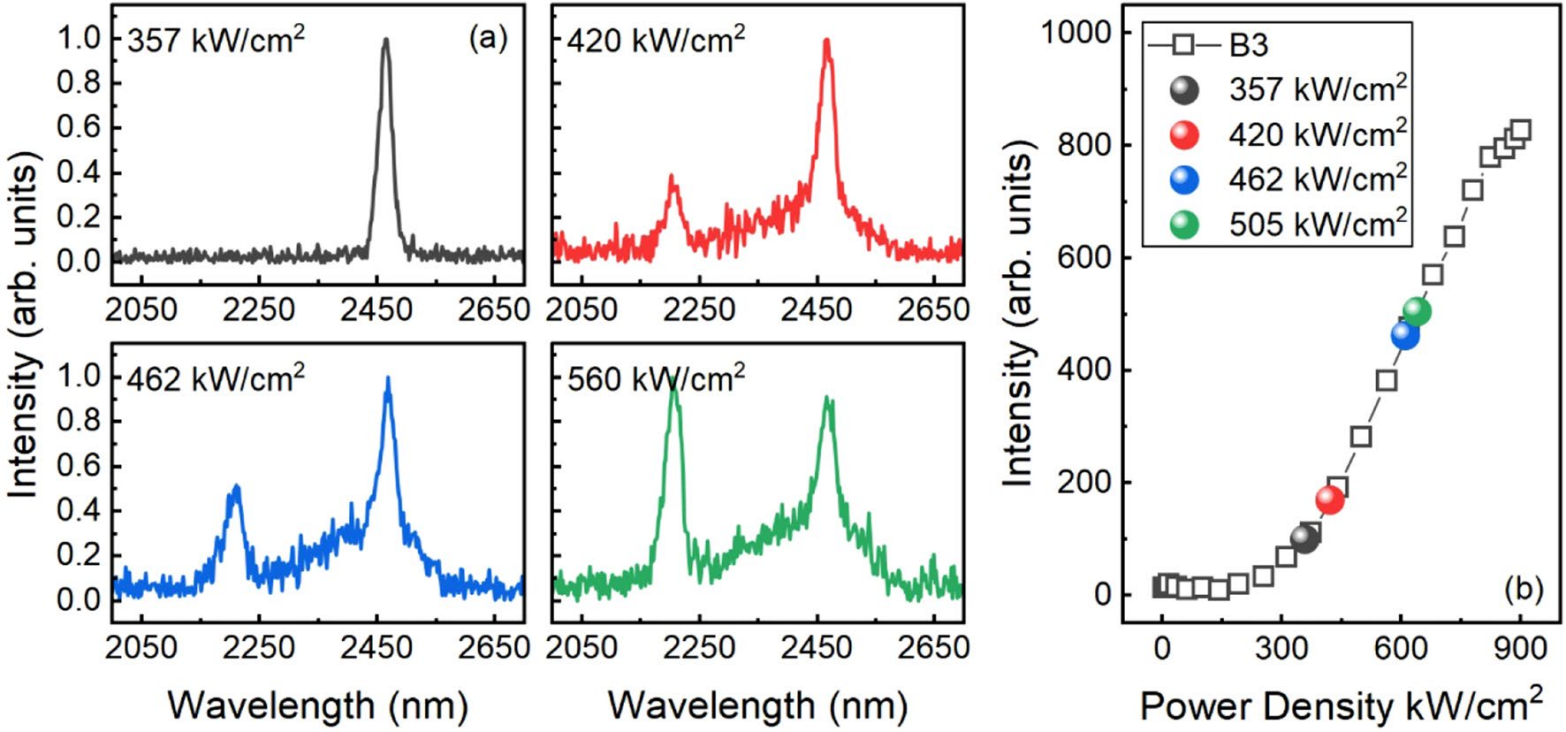
The evolution of dual-wavelength lasing action as a function of pump power for sample B3. (**a**) The lasing spectra at pump power densities of 357, 420, 462, and 505 kW/cm^2^, respectively. (**b**) The L-L dependency for sample B3.

Figure 5a shows the typical pumping power-dependent spectra of sample B3 at 77 K. The MQW lasing was first observed above the threshold at 357 kW/cm^2^, followed by the emerging of GeSn buffer lasing at ~ 420 kW/cm^2^. Two simultaneous lasing peaks that are 273 nm apart at 2187 and 2460 nm are observed. As pumping power keeps increasing, MQW lasing peak exhibits saturation while GeSn buffer peak grows. At 505 kW/cm^2^, the intensity of GeSn buffer peak is higher than that of MQW peak. The corresponding pumping powers can be located in the L-L curve shown in Fig. 5b. The lasing behavior can be explained as the follows: due to the 50-nm-thick bottom SiGeSn barrier serving as partitioning layer, with sufficient high pumping power, each gain region is allowed to draw from its own photogenerated carrier population, leading to the onset of lasing from both regions with dual wavelengths. It has been reported that if the partitioning barrier is too thin, the photons generated at shorter-wavelength region (GeSn buffer) can be reabsorbed at the longer-wavelength region (MQW), and may eventually cease the lasing at shorter-wavelength region.

Unlike the other reports using two sets of MQWs, in our case, the intensity of lasing peak at GeSn buffer keeps increasing, indicating that the two gain regions can be made to operate largely independent of one another. At a higher pumping power, the GeSn buffer region could draw more photogenerated carriers and therefore the stimulated emission is significantly enhanced, resulting in the peak intensity higher than that of MQW. Moreover, since maximum lasing temperatures of GeSn buffer and MQW may be different, the onset of dual-wavelength operation is also dependent on temperature. At this moment further experiments are needed to fully understand the lasing behavior.

## Conclusion

In conclusion, by systemically investigating two groups of samples, we have demonstrated optically pumped dual-wavelength GeSn laser operation at 2187 nm and 2460 nm at 77 K. Two simultaneous lasing regions include a GeSn buffer (bulk) and a SiGeSn/GeSn MQW. A 50-nm-thick bottom SiGeSn barrier serves as partitioning layer, which is sufficiently thick to guarantee two gain regions operating independently. As an all-group-IV laser grown on Si substrate, the GeSn dual-wavelength lasers could well-establish themselves as novel interferometry and terahertz sources with the capability of large-scale integration.

## Data Availability

The data that support the findings of this study are available from the corresponding author upon reasonable request.
